# Comparative analysis of syndromic and PCR-based diagnostic assay reveals misdiagnosis/ overtreatment for trichomoniasis based on subjective judgment in symptomatic patients

**DOI:** 10.1186/s40249-016-0133-x

**Published:** 2016-05-05

**Authors:** Subash Chandra Sonkar, Kirti Wasnik, Anita Kumar, Pratima Mittal, Daman Saluja

**Affiliations:** Dr. B. R. Ambedkar Center for Biomedical Research, University of Delhi, Delhi, 110007 India; Department of Obstetrics & Gynecology, Vardhman Mahavir Medical College and Safdarjung Hospital, New Delhi, 110029 India

**Keywords:** *Trichomoniasis*, Syndromic case management, PCR based diagnosis, Misdiagnosis, Overtreatment

## Abstract

**Background:**

Trichomoniasis, a sexually transmitted disease (STD), is caused by *Trichomonas vaginalis* in both men and women. Screening of trichomoniasis is problematic in resource challenged settings as currently available, inexpensive diagnostic methods are of low sensitivity and/or specificity. In India, National AIDS Control organization (NACO) recommended syndromic case management (SCM) for treatment. The objective of the present study was to compare the utility of the NACO-NACP III Algorithms for STI/RTI treatment used by clinicians with PCR based diagnosis.

**Methods:**

Patients visiting Department of Obstetrics & Gynecology, Vardhman Mahavir Medical College and Safdarjung Hospital, New Delhi from January 2011 to June 2014 were enrolled in the study to compare the diagnostic efficiency of PCR-based assays against SCM. Based on SCM, patients (*n* = 820) were treated with antibiotics using pre-packed STI/RTI kits (sexually transmitted infection/reproductive tract infection; procured by National AIDS Control/State AIDS Control Society (NACO/SACS), Ministry of Health and Family Welfare, Govt of India.) under National AIDS Control Programme (NACP III) for syndromic case management (SCM). Ectocervical dry swab samples were also obtained from these patients and out of that 634 samples were tested by PCR. Total genomic DNA was extracted from these samples and used as template for PCR amplification using *pfoB, gyrA* and *orf1* gene specific primers for diagnosis of *T. vaginalis* (TV), *Chlamydia trachomatis* (CT) and *Neisseria gonorrhoeae* (NG) respectively.

**Results:**

Out of 6000 patients who visited OPD, 820 (14 %) female patients reported vaginal discharge and were recommended antibiotic treatment for one or more pathogens namely, *TV, CT, NG* and *Candida* or for co-infection. On the basis of signs & symptoms and NACO guidelines, the following distribution of various infections was observed: TV (46 %), CT (20 %), coinfection with TV and CT (12 %), coinfection with CT and NG (11 %), coinfection with TV, CT and Candida (7 %) and coinfection with TV and NG (2 %)*.* Others were infected with NG alone (1 %), coinfected with TV and *Candida* (0.4 %) and 0.3 % were coinfected with CT, NG and *Candida*. Based on PCR method, 110 (17 %) women tested positive for one or more of these three pathogens while 524 (83 %) women were negative for any of these three pathogens but could be positive for other STIs not tested in this study. Since all the patients (634) were given antibiotics, we estimate that the over-treatment was 85 % while 524 (83 %) patients were also misdiagnosed by SCM.

**Conclusions:**

The over-treatment and inaccurate diagnosis of pathogens due to subjective judgment based on syndromic approach in symptomatic women is a large economic wastage and may also contribute towards increased resistance. The misdiagnosed patients will also serve as a reservoir for transmission of pathogens to their sexual partner.

**Electronic supplementary material:**

The online version of this article (doi:10.1186/s40249-016-0133-x) contains supplementary material, which is available to authorized users.

## Multilingual abstract

Please see Additional file 1 for translations of the abstract into the six official working languages of the United Nations.

## Background

*Trichomonas vaginalis* (TV) is a flagellated protozoan parasite of the genital tract causing trichomoniasis, a common sexually transmitted infection (STI) affecting men and women [1, 2]. It is one of the “neglected” STDs and approximately 50–60 % infections remain asymptomatic [3, 4]. The clinical symptoms of infection in women include “frothy discharge”, punctate bleeding of the cervix, often referred to as “strawberry cervix”. The less specific symptoms of trichomoniasis include vaginitis, cervicitis, itching, vulval irritation, inflammation, dysuria, lower abdominal pain (LAP) and infection linked with pelvic inflammatory diseases (PID), adverse outcomes of pregnancy and cervical cancer [5–11]. *T. vaginalis* infection also enhances the transmission of human immunodeficiency virus (HIV) infection [5, 8]. Symptoms in men include discharge which may or may not contain significant quantities of lymphocytes or red blood cells (RBC), dysuria, increased urinary frequency or prostatitis [5, 12]. On rare occasions men may have urethral strictures or epididymitis but the vast majority of men with trichomoniasis have no sign or symptoms of infection [13]. The laboratory diagnosis for trichomoniasis includes rapid culture, wet mount, rapid antigen immune capillary, nucleic acid amplification tests (NAATs) and quantitative PCR (qPCR) based assays [14–17]. The developing countries, however, rely on SCM as available rapid tests of trichomoniasis have low sensitivity and specificity [18, 19]. The qPCR requires sophisticated laboratory facilities and qualified personnel with expertise who can perform technically demanding procedures [20, 21]. The culture method, although less expensive and highly specific, is less sensitive, difficult and takes several days. As test results cannot be made available immediately, delay and/or failure of the patient to come back to collect the test report, impede the treatment initiation [20–22]. Such patients continue to spread the disease to their sexual partners by serving as the reservoirs [23–25]. For these reasons, the SCM of trichomoniasis remains the most feasible option for clinician in low resource settings. SCM of the disease is based on the identification of signs and symptoms that characterize standard clinical symptoms of the disease so as to predict the likelihood that a patient is infected with a specific pathogen [17, 18]. Based on the prediction of infection, appropriate treatment is prescribed at the first visit itself rather than deferring the treatment until an accurate and confirmed diagnosis is obtained. In the absence of a proper, inexpensive diagnostic assay in the resource poor settings, the syndromic approach takes on greater relevance in controlling the transmission of STIs and its sequel. However, despite these advantages, there are several limitations associated with SCM [26]. As SCM relies on subjective judgment, it may result in over diagnosis/overtreatment in patients infected with microbes other than *T vaginalis*. This may also contribute towards the development of drug resistance against other STIs [27–29]. Another challenge for controlling STIs by syndromic approach is mixed infection by more than one pathogen with similar clinical presentations [30]. Due to the lack of trained personnel, inadequate laboratories and infrastructure in several parts of the developing countries, information regarding the profile of STIs relies essentially on self-reported or physician-diagnosed STI and hence prevalence amongst asymptomatic individuals is unavailable. This contributes to the lack of treatment of asymptomatic patients, resulting in the spread of the disease to their sexual partners. In fact, there are no standard protocols and/or line of treatment that is prescribed for SCM of trichomoniasis in India. As a result, there is no consensus on the performance of SCM of *T. vaginalis* and reports from different regions carried out on small groups of patients, fail to provide a clear and consistent evaluation [31–34]. Thus, there is an urgent need to evaluate the validity of the SCM of *T vaginalis* infection. Here we report the results of a detailed comparative study on syndromic and PCR-based laboratory assay for diagnosis of *T vaginalis* using 634 clinical samples collected from the Department of Obstetrics & Gynecology, Vardhman Mahavir Medical College (VMMC) and Safdarjung Hospital, New Delhi, India.

## Methods

### Ethics statements

The study was carried out as per the institutional ethical guidelines and approval (ACBR No: F-50-2/Eth.com/ACBR/11/2107 and VMMC and Safdarjung Hospital No: 47-11-EC/30/51). Informed written consent from all participants involved in the study was obtained. The results of the study did not influence the treatment.

### Enrollment of patients

The patients visiting Department of Obstetrics & Gynecology of VMMC and gynecology OPD of Safdarjung Hospital, New Delhi, from January 2011 to June 2014 were enrolled in this study. Out of 6 000 patients who visited OPD during this period, ectocervical swab samples (*n* = 634) were collected from non-pregnant women (18 years to ≥ 56 years of age) seeking diagnosis and treatment of vaginal discharge syndrome (VDS) or PID. Individuals who were already on antibiotics, pregnant, unmarried and females below 18 years of age were excluded. To confirm non-pregnancy every patient coming to Gynecology OPD was asked about her last menstrual period. If she was overdue, pregnancy test (urine based) was conducted in the OPD on the same day.

### Specimen collection and processing

A thorough clinical examination including the speculum examination was done for lesions, warts, ectopic growth and vaginal/cervical discharge by the attending clinician. Ectocervical swab samples were collected and placed in the empty vial (dry swabs) and were kept frozen at −20 °C until use. Dry swab was incubated in Phosphate Buffered Saline (PBS, 1 mL) for 10 min at 4 °C, mixed by vortexing thoroughly to disperse the sample and squeezed. The sample (400 μl) was centrifuged at 11 000 × *g* at 4 °C for 10 min and the cell pellet was suspended in 40 μl of PBS followed by centrifugation at 11 000 × *g* at 4 °C for 5 min. Total genomic DNA was isolated essentially as described earlier from our laboratory for other pathogens [35, 36]. Briefly, the cell pellet was suspended in 40 μl of 1X lysis buffer containing 50 mM Tris–HCl (pH 7.5), 1 % Triton X-100, proteinase K (250 μg/ml). The cell suspension was incubated at 65 °C for 45 min to lyse the cells, followed by boiling for 10 min at 95 °C after addition of DTT (1 mM). Sample was then snap-chilled for 5 min followed by centrifugation at 8 000 × *g* at 4 °C for 10 min. To the supernatant, 50 μl of phenol-chloroform-isoamyl alcohol (25:24:1) was added and the aqueous phase containing DNA was collected. The nucleic acids were precipitated by adding 1/10 volume of 3 M sodium acetate (pH 5.2), glycogen (1μl20mg/ml) and 2.5 volumes of 100 % ethanol. After incubating at 20 °C for 1-2 h, samples were centrifuged at 11 000 × *g* at room temperature for 10 min and the nucleic acids pellet was washed with 70 % chilled ethanol by centrifugation at 11 000 × *g* at room temperature for 5 min. The pellet was air dried at 55 °C before suspending in either 50 μl of TE buffer, pH 7.0 (10 mM Tris HCl, 1 mM EDTA) or in DNase- and RNase-free water (Sigma-Aldrich, St. Louis, Mo. W4502). The nucleic acid samples were stored at 4 °C till further use as template DNA for PCR assay and clinical evaluation.

### PCR amplification

All the clinical samples were analyzed for the presence of *T. vaginalis* using in-house developed *pfoB* PCR assay. The in-house developed assay was evaluated by composite reference standard (CRS) method and found to be highly sensitive, 94.44 (*CI* 95 % 81.30–99.16) and specific, 99.72 (*CI* 95 % 98.44–99.95), it can detect up to 25 fg genomic DNA with remarkable NPV, PPV and Likelihood ratio (Sonkar et, al. manuscript submitted) and Indian patent filed and published, Patent application No. 1098/DEL/2013A, publication date: 03/01/2014) [36]. The diagnosis of *C. trachomatis* and *N. gonorrhoeae* was carried out using PCR based assay developed earlier [35, 37]. Briefly, PCR amplification was carried out in 25 μl reaction mix containing 1X Taq DNA Polymerase, buffer (50 mM KCl, 10 mM Tris-HCl pH 8.3, 1.5 mM MgCl_2_), 200 μM each of the four deoxyribonucleoside triphosphates (dNTPs) (Bangalore Genei India Pvt. Ltd.), 5pmoles each of forward and reverse primer, 5 μl of total genomic DNA isolated from clinical sample, 2.0 U of Taq DNA Polymerase (Bangalore Genei India Pvt. Ltd.). Each set of PCR assays included a negative control (sterile water instead of DNA sample) and a positive control (1 ng of purified genomic DNA of *T. vaginalis)*. Amplification of *T. vaginalis* was performed in the thermal cycler (I cycler, BioRad, USA) using the following conditions; 96 °C for 5 min for initial denaturation, 35 cycles of 96 °C for 20 s, 56 °C for 30 s, 72 °C for 25 s and final extension at 72 °C for 7 min. The amplification of *C. trachomatis* and *N. gonorrhoeae* was as per published protocol [35, 37].

### Detection of amplified products

The amplicons in 25 μl were analyzed by electrophoresis on 2.0 % agarose gel and visualized by ethidium bromide staining and illumination with UV light (LumG Aplegen, USA). The amplicons from 10 % of the positive samples were eluted using a DNA elution kit (MDI membrane Technologies, India), according to the manufacturer′s instructions and sequenced using forward PCR primers with Big Dye terminator cycle sequencing kit on 377A autosequencer (Applied Biosystems, California, USA). The DNA sequence of the amplified product was compared to that of known sequence of pfoB, *orf1* and *gyrA* gene for *T. vaginalis, N. gonorrhoeae* and *C. trachomatis* respectively in the GenBank databases using the BLASTn program (http://blast.ncbi.nlm.nih.gov) to determine the percent identity.

### Statistical analysis

The data was plotted and analyzed using the statistical software package SPSS version 20.

## Results

**Fig. 1:**
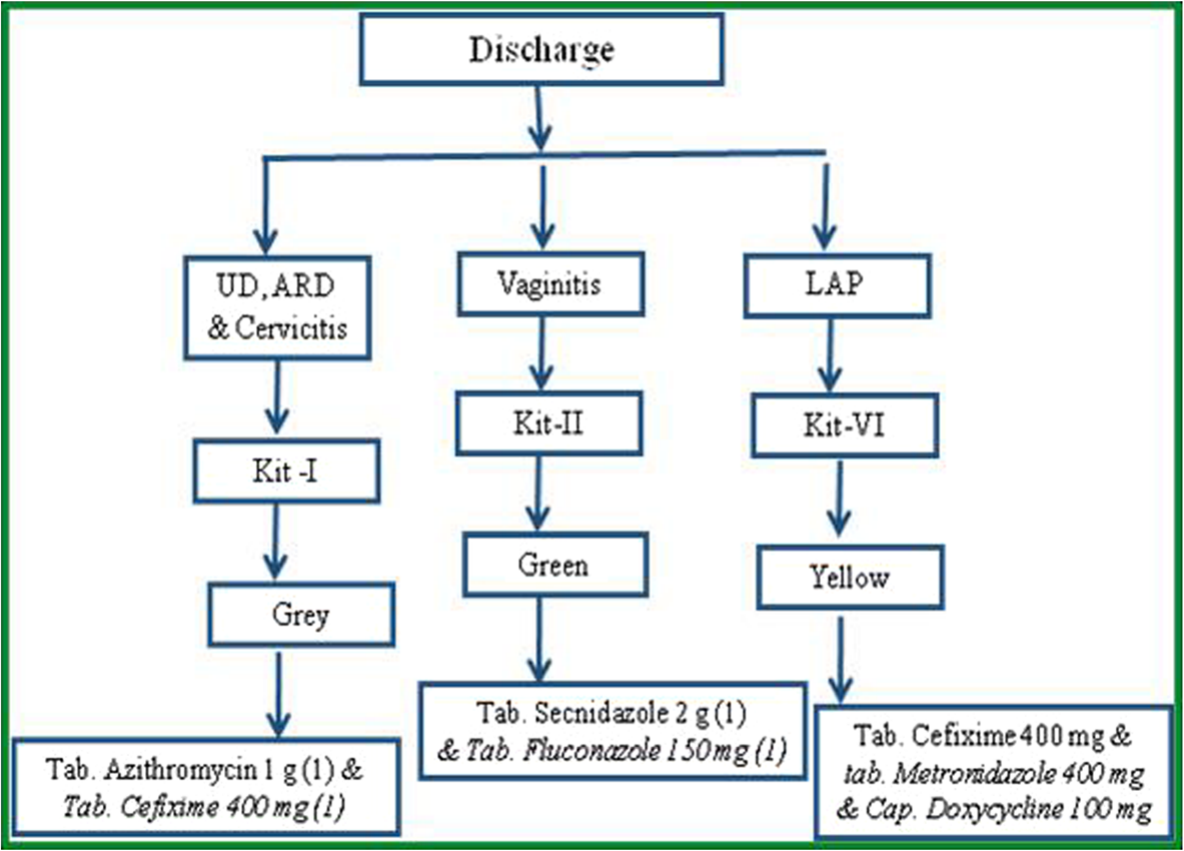
Proposed pre-packed STI/RTI kits under NACP III for syndromic case management procured by NACO/SACS, Ministry of Health and Family Welfare, August 2007 (NACO-NACP III Algorithms for STI/RTI used by Clinicians)

**Fig. 2:**
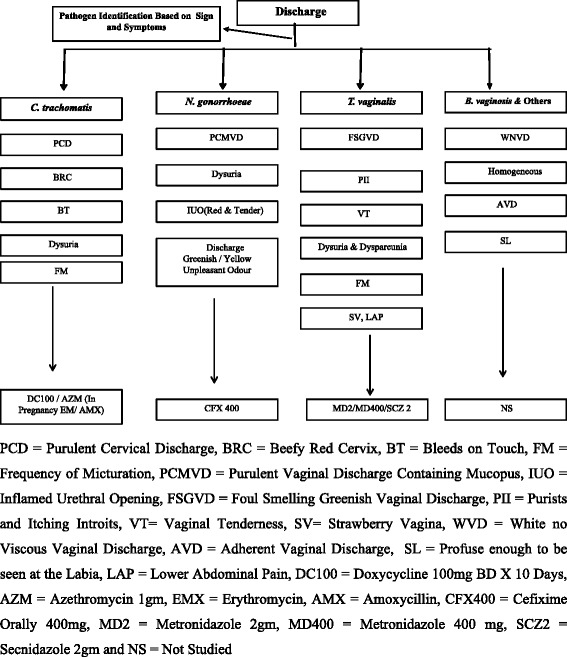
NACO-NACP III Algorithms for STI/RTI used by Clinician

**Fig. 3:**
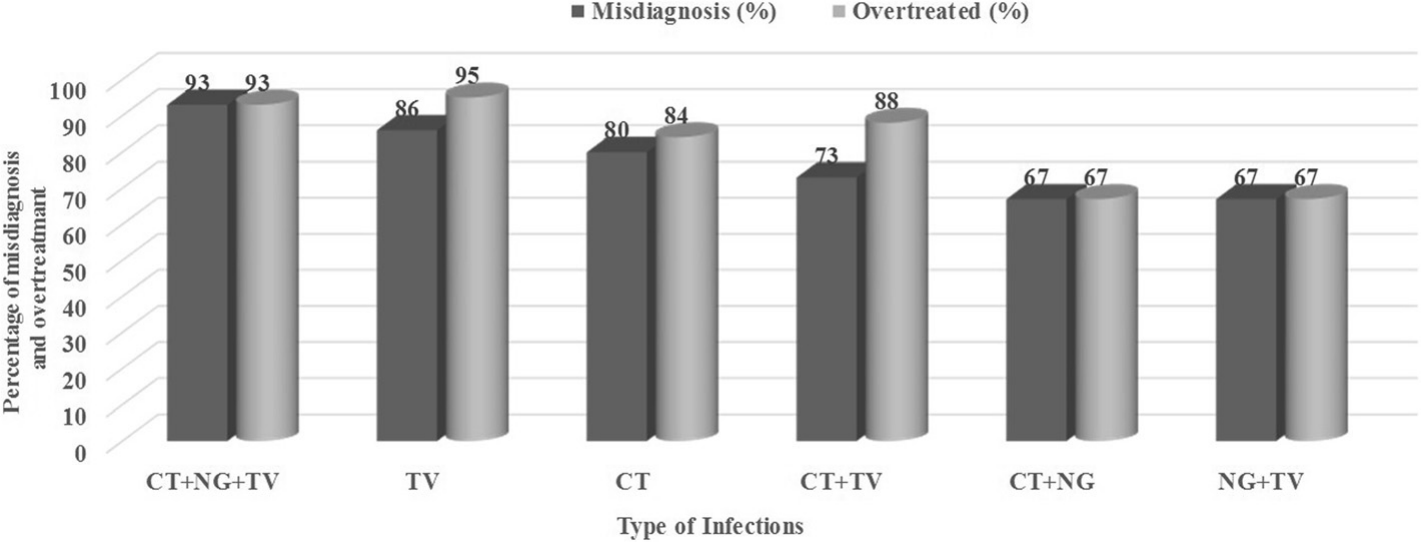
Bar diagram showing percent misdiagnosis and overtreatment based on comparison of SCM and PCR assays. The patients were classified as infected for TV, CT and NG based on definite diagnosis by PCR and symptom based diagnosis by SCM (See Table 1). All patients were given antibiotics based on SCM. Overtreatment and misdiagnosis by SCM for infection and coinfection with TV, CT and NG were calculated by comparing with PCR results

A clinic-based study was carried out in association with the Department of Obstetrics & Gynecology Department of VMMC and Safdarjung Hospital gynecology OPD from January 2011-June 2014. During this period 6 000 patients visited the OPD out of which 820 (14 %) female patients with vaginal discharge suspected of infection with *T. vaginalis, C. trachomatis, N. gonorrhoeae* and *Candida* were recruited based on SCM (see Figs. 1 and 2). These 820 patients were recommended pre-packed STI/RTI antibiotic treatment kits procured from NACO/SACS, Ministry of Health and Family Welfare, Govt of India, under the NACP III for disease management. Ectocervical dry swab samples were also obtained from these 820 patients out of which 634 patients were scored in this study. The other samples could not be included as the symptom descriptions and demographic data were incomplete. Out of 634 samples that were considered positive for one or more pathogens using SCM approach, PCR based diagnostic assay showed only 110 samples to be positive for one of the pathogens, *C. trachomatis* (CT), *N. gonorrhoeae* (NG) and *T. vaginalis* (TV) or coinfections (Table 1). Since all the patients (*n* = 634) were given antibiotics on the basis of SCM, our results show that a large proportion (85 %) of the patients were over treated due to lack of accurate and confirmed diagnosis. When we analyzed the coinfection status of these samples for TV, CT and NG using SCM approach, it was observed that 12 % patients were coinfected with TV, CT and NG while 11 % were coinfected with CT and NG and 7 % with CT and TV (Table 1). According to SCM, infection with NG was low in general (less than 1 %) and most of the patients were co-infected with TV (1 % of total patients). To differentiate between *T. vaginalis* and *Bacterial vaginosis* amongst the suspected cases with vaginal discharge (*n* = 634), the color, smell and viscosity of discharge were the main differentiating symptoms as per NACO-NACP III Algorithms for STI/RTI (Fig. 2). Based on the symptoms listed in Fig. 2, trichomoniasis was reported in 289 patients out of 634 (46 %) whereas based on PCR assay only (2 %) patients suffered from *T vaginalis* infection alone and many patients had coinfection (see Table 1). Similarly, infection with CT by PCR assay amounts to only 7 % (42/634) while based on SCM infection by CT is 20 % (128/634). Likewise large proportions of false positives were also observed by SCM for co-infections when compared with PCR based detection method (see Table 1). Coinfection of TV and *Candida* and coinfection of CT, NG and *Candida* could not be compared with PCR in the present study*.* Thus a huge fraction of patients (83 %) were inaccurately diagnosed using SCM for the three pathogens tested in this study and all these patients were also given antibiotics leading to huge overtreatment (see Fig. 3 and Table 1). Amongst these misdiagnosed cases of trichomoniasis by SCM, 6 and 4 %, were actually infected with CT and NG respectively while 2 % & ≤ 1 % were co-infected with CT and CT+ NG respectively (Table. 1). It is pertinent to mention here that a substantial population of patients remained undiagnosed for the actual infecting microbe.

**Table 1:**
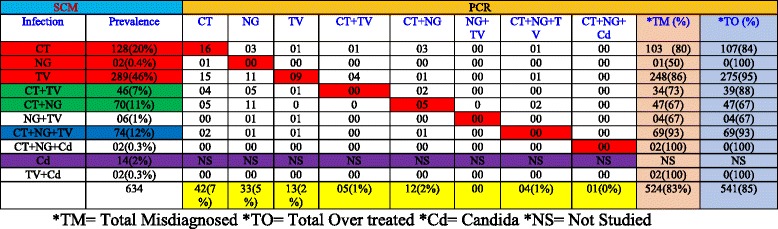
Tabular presentation of infection using SCM and PCR assays

**Fig. 4:**
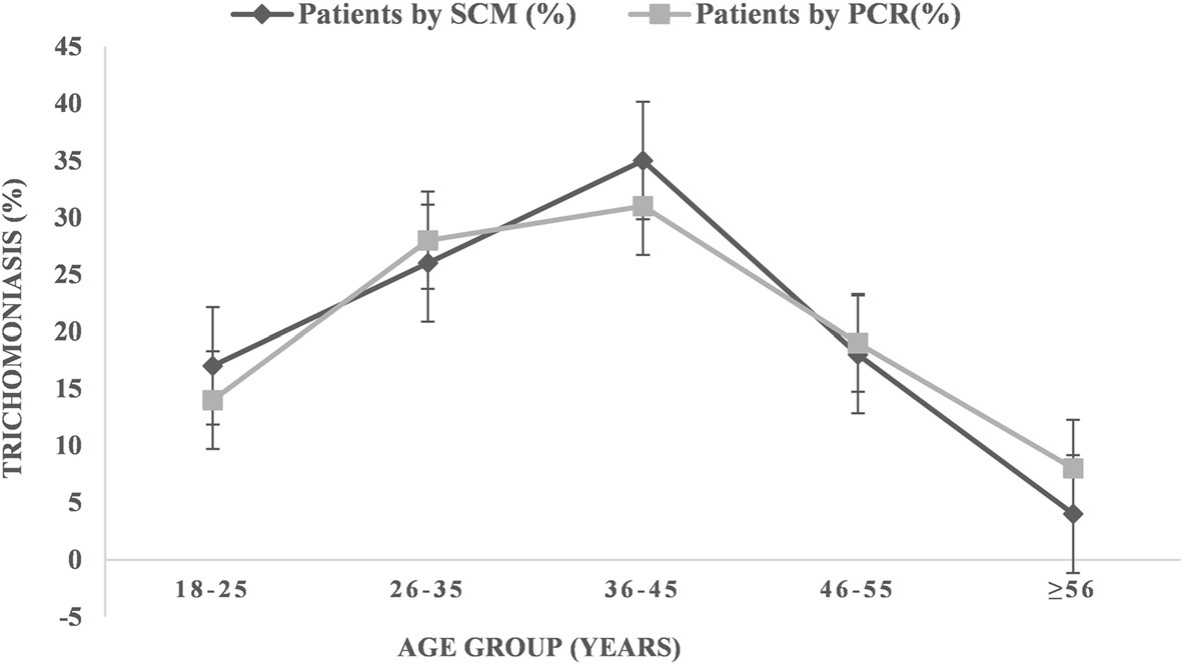
Age wise distribution of *Trichomonas vaginalis* infection among symptomatic women visiting the OPD of Obstetrics & Gynecology

**Fig. 5:**
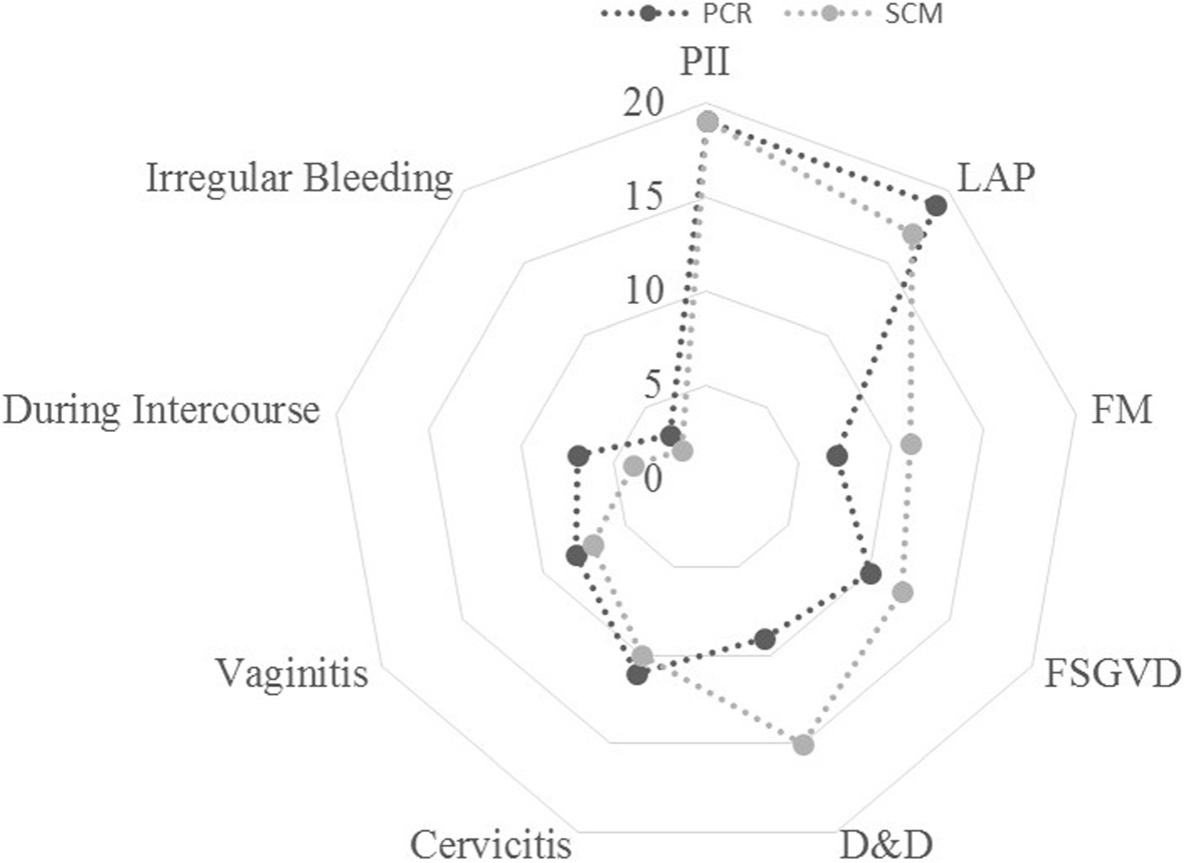
Association of different symptoms in patients with *Trichomonas vaginalis.* Different symptoms observed in patients infected with *T. vaginalis* based on SCM or PCR were plotted. As observed, none of the symptoms is uniquely associated with *T. vaginalis* infection as maximum association of any symptom found in the patients is less than 20 % (PII = Purists & Itching Introits, LAP = Lower Abdominal Pain, FM = Frequency of Micturition, FSGVD = Foul Smelling Greenish Vaginal Discharge, D&D = Dysuria & Dyspareunia)

In order to find out which reproductive age group is most vulnerable to *T. vaginalis* infection, the samples collected from patients in different age groups 18–25, 26–35, 36–45, 46–55 and >55 years were compared based on the diagnosis by PCR and also SCM. The distribution of *T. vaginalis* infection complaints among all the age groups was similar based on SCM as well PCR as reported by others [18, 38, 39]. In both PCR and SCM, infection with *T. vaginalis* is most prevalent (25–35 %) in the age groups of 26–35 years and 36–45 years (see Fig. 4). Using the clinical symptoms for syndromic management, we also tried to find out how strongly the symptoms are associated with infection. Amongst these samples and based on syndromic approach, we observed significant association of *Trichomonas* infection with patients having purists and itching (19 %), lower abdominal pain (17 %), dysuria and dyspareunia (15 %), foul smelling discharge (12 %), frequency of micturition (11 %), vaginitis (10 %), cervicitis (7 %), irregular bleeding (5 %) and pain during intercourse (4 %). Using PCR based diagnostic assay the percent symptoms associated with *T. vaginalis* infected patients were; lower abdominal pain (21 %), itching (18 %), foul smelling discharge (14 %), vaginitis (12 %), dysuria (11 %), cervicitis (9 %), pain during intercourse (7 %), frequency of micturition (5 %) and irregular bleeding (3 %). As is evident from these results, irrespective of the diagnostic method used, the pattern of symptoms remain similar in the infected population, however, none of the symptoms was significantly or categorically specific to the disease (see Fig. 5). The maximum association of any symptom was about 20 % whether it is itching or lower abdominal pain.

## Discussion

The burden of trichomoniasis in poor resource settings and high-risk groups of industrialized settings in developing countries is soaring due to lack of accurate and confirmed diagnosis [40]. SCM of the disease has several limitations including overtreatment of symptomatic patients. In addition, high rate of asymptomatic infection in male partners of infected females and subsequent re-infection have significant implications in the control and management program for STI [25, 41]. In India, Government hospitals and peripheral laboratories continue to characterize and manage the STI using syndromic approach [42–46]. The existing standard protocol used for detection is pelvic examination (adolescent women), microscopic detection and wet mount in clinical setups [47–49]. Since wet mount is reported to be approximately 50–60 % sensitive, it limits accurate and confirmed detection of trichomoniasis [50–54]. The Point of Care test, such as OSOM, InPouchTV, lateral flow strip test, TMA and other rapid tests (vaginal pH and the presence of amines) are also not sufficiently sensitive for confirmed detection [21, 55–62]. The introduction of PCR based diagnostics has enhanced our understanding of the epidemiology of *T. vaginalis*. However, due to high cost of imported kits, the NAAT-based assays are not commonly used in India. The present study was undertaken to establish the need of diagnosis-based treatment of trichomoniasis by comparing the percentage of patients who were genuinely infected, as evident by PCR based diagnostics, and those diagnosed based on symptoms using SCM approach. Our results clearly establish that the SCM is imprecise and results in enormous overtreatment of patients. This may contribute to the development of resistance to common antibiotics.

The effective treatment available against trichomoniasis makes it one of the most curable STIs but undiagnosed and untreated infections may serve as a reservoir for spreading the infection and opportunistic infection by other STIs. The long term serious consequences including pelvic inflammatory disease, preterm births or low-birth-weight of infants are also associated with poor diagnosis. WHO developed a simplified tool (a flowchart or algorithm) to guide health workers in the implementation of syndromic management of STI for women with symptoms of vaginal discharge and/or lower abdominal pain (see Figs. 1 and 2). However, it is important to recognize the limitations of the vaginal discharge algorithms, particularly in the management of cervical infection (Trichomoniasis, gonococcal and Chlamydial infections). In the present study, we observed a large number of false positives based on symptoms and all these patients were given treatment with one or more antibiotics as per pre-packed STI/RTI kits procured by NACO/SACS, Ministry of Health and Family Welfare, Govt of India under NACP III for diseases management. The problem with syndromic management is not only misdiagnosis but also overtreatment. Moreover, often the patient is not given treatment for the actual causative pathogen. The management of trichomoniasis based on symptoms is therefore highly unreliable because the spectrum of infection is broad and other STIs have similar clinical presentations and it is difficult to differentiate and characterize the causative agent by the routine clinical observations (Fig. 5). In general, but especially in low prevalence settings and in adolescent females, endogenous vaginitis rather than STI is the main cause of vaginal discharge [51]. While attempts have been made to increase the sensitivity and specificity of the vaginal discharge algorithm for the diagnosis of cervical infection through the introduction of an appropriate, situation-specific risk assessment, remain low in our studies as we did not observe any symptom that was specifically associated with all TV patients. The maximum association of any symptoms was 20 % which is too low an association to be used to classify the cause of infection. Moreover, some of the risk assessment questions based on demographics, low income, illiteracy, religion, occupations, marital status and pregnancy tend to incorrectly classify too many adolescents to be at risk of infection [6, 63–64]. Thus SCM is unsuitable for routine clinical diagnosis of trichomoniasis even in resource poor countries. Consequently, there is a need to identify the main trichomoniasis risk factors in women in the local population and tailor the risk assessment accordingly. It is equally important to develop and evaluate rapid, specific and inexpensive tests to diagnose and control trichomoniasis. The gold standard method for detection of trichomoniasis in women is culture of vaginal swab specimens. The drawback of culture is, requirement of long duration of incubation for the growth of the organism, need for expert microbiologist and low sensitivity compared to nucleic acid amplification tests (NAATs) [15, 65]. The traditional clinical diagnostic methods fail to identify more than one half of mixed infections which if left untreated can result in adverse pregnancy outcomes and exacerbated risk for both acquisition and transmission of HIV [6, 66–69]. The currently available POC diagnostic assays for *T. vaginalis* are promising but not as sensitive as NAAT based assays [70–74]. Undoubtedly, there is an urgent need to reduce the cost and complexity of NAAT-based assay so that they can be performed in resource poor settings in developing countries.

## Conclusion

The study provides evidence to show that in spite of several advantages of SCM of trichomoniasis, definite diagnosis must be carried out before starting the treatment. SCM purely relies on subjective judgment leading to misdiagnosis and overtreatment in infected patients. It may results in developing antibiotic resistance. Moreover, over-treatment and inaccurate diagnosis, based on syndromic approach, is a large economic wastage.

## Additional file

Additional file 1: Multilingual abstract in the six official working languages of the United Nations. (PDF 421 kb)

## Abbreviations

NAAT: nucleic acid amplification test
NACO: National AIDS Control Organization
NACP: National AIDS Control Programme
NPV: negative predictive value
PBS: phosphate buffered saline
PCR: polymerase chain reaction
PPV: positive predictive value
RTI: reproductive tract infection
SACS: State AIDS Control Society
SCM: syndromic case management
STD: sexually transmitted disease
STI: sexually transmitted infection
