# Supplementary material for: Comparative analysis of syndromic and PCR-based diagnostic assay reveals misdiagnosis/ overtreatment for trichomoniasis based on subjective judgment in symptomatic patients
Source: Infect Dis Poverty. 2016 May 5;5:42. doi: 10.1186/s40249-016-0133-x (PMC4857337; doi:10.1186/s40249-016-0133-x)
Supplement: Additional file 1: — Multilingual abstract in the six official working languages of the United Nations. (PDF 421 kb) [file 40249_2016_133_MOESM1_ESM.pdf]

Translation of the abstract into the five official working languages of the United Nations

## التحليل المقارن والمتلازمي واختبار التشخيص القائم على تفاعل البلمرة المتسلسل يكشف التشخيص الخاطئ/فرط اللجوء إلى المعالجة في داء المشعرات استنادا إلى الحكم الموضوعي في المرضى الذين يعانون من أعراض

سوباش شاندرا سونكار، كيرتي واسنيك، أنيتا كومار، براتيما ميتال، دامان سالوجا

**ملخص**

**خلفية:** المشعرات المهبلية هو مرض ينتقل عن طريق الاتصال الجنسي مسببا داء المشعرات في كل من الرجال والنساء. فحص داء المشعرات هو مشكلة في المناطق التي تنقصها الموارد حاليا، وطرق التشخيص رخيصة ذات حساسية و / أو خصوصية منخفضة. في الهند، أوصت المنظمة الوطنية لمكافحة الإيدز باستخدام الإدارة المتلازمية للحالات (SCM) للعلاج. وكان الهدف من هذه الدراسة مقارنة فائدة الإصدار الثالث من إجراءات المنظمة الوطنية لمكافحة الإيدز-البرنامج الوطني لمكافحة الإيدز لعلاج الأمراض المنقولة جنسيا/عدوى الجهاز التناسلي التي يستخدمها الأطباء في التشخيص القائم على تفاعل البلمرة المتسلسل.

**الطرق:** ألحق مرضى قسم التوليد وأمراض النساء في كلية فاردمان ماهافير الطبية ومستشفى سافدارجونج في نيو دلهي من يناير 2011 إلى يونيو لعام 2014 في دراسة لمقارنة كفاءة التشخيص للفحوص القائمة على تفاعل البلمرة المتسلسل مقابل الإدارة المتلازمية للحالات. وبناء على الإدارة المتلازمية للحالات، عولج المرضى (ن = 820) من العدوى المنقولة جنسيا / عدوى الجهاز التناسلي باستخدام مضادات حيوية معبأة سلفا اشترتها المنظمة الوطنية لمكافحة الإيدز/ الجمعية الحكومية لمكافحة الإيدز، ووزارة الصحة ورعاية الأسرة، وحكومة الهند.) ضمن البرنامج الوطني لمكافحة الإيدز (البرنامج الوطني الثالث لمكافحة الإيدز) للإدارة المتلازمية للحالات. وقد تم الحصول على عينات مسحة جافة من ظاهر عنق الرحم أيضا من هؤلاء المرضى تم اختبار 634 عينة من المرضى باستخدام تفاعل البلمرة المتسلسل. تم استخراج الحمض النووي الجيني الكلي من هذه العينات واستخدامها كقالب لتضخيم تفاعل البلمرة المتسلسل باستخدام البادئات المحددة الجينات pfoB وgyrA وorf1 لتشخيص المشعرات المهبلية والمتدثرة الحثرية والنيسرية البنية على التوالي.

**النتائج:** من 6000 مريضا زاروا العيادات الخارجية، أفاد 820 (14%) من المرضى الإناث بوجود إفرازات المهبلية لديهن وأوصى بعلاجهن بالمضادات الحيوية لمدة سنة أو أكثر من مسببات الأمراض وهي، المشعرات المهبلية والمتدثرة الحثرية والنيسرية البنية والكلاميديا أو الإصابات المشتركة. واستنادا على العلامات والأعراض والمبادئ التوجيهية للمنظمة الوطنية لمكافحة الإيدز، لوحظ التوزيع التالي للإصابة بالأمراض المعدية المختلفة: المشعرات المهبلية (46%)، المتدثرة الحثرية (20%)، عدوى مشتركة للمشعرات المهبلية والمتدثرة الحثرية (12%)، عدوى مشتركة للمتدثرة الحثرية والنيسرية البنية (11%)، عدوى مشتركة للمشعرات المهبلية، والمتدثرة الحثرية والكلاميديا (7%) وعدوى مشتركة للمشعرات المهبلية والنيسرية البنية (2%). آخرون مصابون بالنيسرية البنية وحدها (1%)، المصابون المشعرات المهبلية والكلاميديا (0.4%) وكان المصابون المتدثرة الحثرية، النيسرية البنية والكلاميديا0.3%. واستنادا إلى طريقة تفاعل البلمرة المتسلسل، كان 110 (17%) من النساء مصابات بواحد أو أكثر من هذه الجراثيم الثلاثة في حين كانت 524 (83%) من النساء غير مصابات بأي من هذه الجراثيم الثلاثة ولكن يمكن أن تكون مصابة بأنواع العدوى المنقولة جنسيا الأخرى التي لم تختبر في هذه الدراسة. وحيث أن جميع المرضى البالغ عددهم (634) عولجوا بالمضادات الحيوية، نقدر نسبة الإفراط في العلاج لتكون 85% بينما بلغت نسبة تشخص خطأ (83%) ما يعادل 524 من المرضى أيضا بسبب الإدارة المتلازمية للحالات.

**الاستنتاجات:** الإفراط في العلاج والتشخيص غير الدقيق لمسببات الأمراض بسبب الحكم الموضوعي المستند على اتباع النهج المتلازمي مع النساء اللائي يعانين من أعراض هو هدر اقتصادي كبير ويمكن أن يسهم أيضا في زيادة مقاومة المرض. والمرضى الذين يتم تشخيص إصابتهم بشكل خاطئ يكونوا أيضا مسبب لانتقال الأمراض إلى الشريك الجنسي.

Translated from English version into Arabic by Mahmoud Sami, through

TRANSLATORS
WITHOUT BORDERS

## 综合症和PCR诊断的比较分析揭示对有症状患者主观判断导致滴虫病的误诊/过度治疗

Subash Chandra Sonkar, KirtiWasnik, Anita Kumar, Pratima Mittal, Daman Saluja

**摘要**

**引言：**滴虫病是一种性传播疾病(STD)，由阴道毛滴虫引起。当前筛查滴虫病面临许多困难，廉价诊断方法的灵敏性和/或特异性较低。在印度，国家艾滋病控制组织(NACO)推荐使用综合症病例管理方法(SCM)治疗滴虫病。本研究的目标在于比较在临床医生治疗 STI/RTI 时 NACO-NACPIII准则和 PCR 诊断的有效性。

**方法：**本研究收集了 2011 年 1 月至 2014 年 6 月新德里 VardhmanMahavir 医学院和 Safdarjung 医院妇产科的患者样本，比较 PCR 检测和 SCM 的诊断效率。基于 SCM，根据国家艾滋病控制项目(NACPIII)，820 例病例采用用于治疗性传播疾病/生殖途径传播疾病的预包装 STI/RTI 试剂盒 (由印度卫生和家庭福利部，国家艾滋病控制/邦艾滋病控制协会(NACO/SACS)提供)进行抗生素治疗。同时获得上述病例的宫颈拭子样品，其中 634 份用于 PCR 检测。从上述样品中抽提全基因组 DNA 作为 PCR 扩增的模板，分别使用 *pfoB*、*gyrA* 和 *orf1* 基因的特异引物来鉴别阴道毛滴虫(*T. vaginalis*，*TV*)，沙眼衣原体(*Chlamydia trachomatis*，*CT*)和淋病奈瑟菌(*Neisseria gonorrhoeae*，*NG*)。

**结果：**访问 OPD 的 6 000 例患者中，820 例(14%)女性患者出现阴道分泌物，被推荐使用抗生素治疗 *TV*、*CT*、*NG* 和念珠菌中的一种或合并感染。根据体症、症状及 NACO 准则，感染情况如下：TV 感染占 46%，CT 感染占 20%，TV 和 CT 合并感染占 12%，CT 和 NG 合并感染占 11%，TV、CT 和念珠菌合并感染占 7%，TV 和 NG 合并感染占 2%，NG 感染占 1%，TV 和念珠菌合并感染占 0.4%，CT、NG 和念珠菌合并感染占 0.3%。PCR 检测病例中，其中 110 例(17%)女性患者一种或多种病原体检测为阳性，其余 524 例(83%)检测为阴性，不排除本研究中未涉及的其他 STIs 检测为阳性。因为所有 634 例病例均进行抗生素治疗，估计过度治疗率为 85%，而 524 例(83%)患者被误诊。

**结论：**对有症状患者从综合症着手进行主观判断导致的病原体过度治疗和错误诊断造成巨大的经济学损失，同时会促进耐药性增强。误诊患者也可能作为传染源将病原体传播给其性伴侣。

Translated from English version into Chinese by Song Peng, edited by Yang Pin, through

CHINESE CENTER FOR DISEASE CONTROL AND PREVENTION
NATIONAL INSTITUTE OF PARASITIC DISEASES

**L'analyse comparative du diagnostic syndromique et par RCP révèle des erreurs de diagnostic et surtraitements de la trichomonase sur la base du jugement subjectif des patients symptomatiques**

Subash Chandra Sonkar, Kirti Wasnik, Anita Kumar, Pratima Mittal, Daman Saluja

**Résumé**

**Contexte :** La trichomonase à *Trichomonas vaginalis* est une maladie sexuellement transmissible (MST) qui affecte les deux sexes. Son dépistage est difficile lorsque les ressources disponibles sont limitées car les méthodes de diagnostic les moins coûteuses actuellement sont peu sensibles et/ou peu spécifiques. L'organisation nationale indienne de lutte contre le SIDA (NACO) recommande une gestion de cas syndromique (GCS) pour le traitement. L'objectif de la présente étude était de comparer l'utilité des algorithmes NACO-NACP III utilisés par les cliniciens pour le traitement des IST/IAR (infections sexuellement transmissibles/infections de l'appareil reproducteur) avec le diagnostic par RCP.

**Méthodes :** Des patientes venues en consultation dans le département de gynécologie-obstétrique à la Faculté de médecine Vardhman Mahavir et à l'Hôpital Safdarjung de New Delhi ont été enrôlées dans l'étude entre janvier

2011 et juin 2014 afin de comparer l'efficacité des essais par RCP et de la gestion de cas syndromique. Dans la GCS, les patientes (n=820) ont été traitées avec des antibiotiques fournis sous forme de kits IST/IAR par National AIDS Control et la State AIDS Control Society (NACO/SACS), relevant du Ministère de la Santé et de la Famille du gouvernement indien, dans le cadre du Programme national de lutte contre le SIDA (NACP III). Des frottis exocervicaux secs ont été prélevés sur ces patientes et 634 de ces échantillons ont été testés par RCP. L'ADN génomique total en a été extrait et utilisé comme modèle pour l'amplification en RCP avec des amorces spécifiques des gènes *pfoB, gyrA* et *orf1* pour le diagnostic de *T. vaginalis* (TV), *Chlamydia trachomatis* (CT) et *Neisseria gonorrhoeae* (NG), respectivement.

**Résultats :** Sur 6000 patientes vues en consultations externes, 820 (14 %) signalaient des écoulements vaginaux et un traitement antibiotique leur a été recommandé contre un ou plusieurs agents pathogènes (*TV, CT, NG* et *Candida* ou co-infection). Sur la base des signes et symptômes et des directives de la NACO, la distribution des différentes infections était la suivante : TV (46 %), CT (20 %), co-infection par TV + CT (12 %), co-infection par CT + NG (11 %), co-infection par TV, CT et *Candida* (7 %) et co-infection par TV et NG (2 %). D'autres patientes étaient infectées seulement par NG (1 %), ci-infectées par TV et *Candida* (0,4 %) ou co-infectées par CT, NG et *Candida* (0,3 %). Sur la base de l'essai de RCP, 110 (17 %) des femmes étaient positives pour un ou plusieurs de ces trois pathogènes mais 524 (83 %) étaient négatives pour tous les trois, quoiqu'elles aient pu être positives pour d'autres IST non recherchées dans l'étude. Dans la mesure où toutes les patientes (634) ont reçu des antibiotiques, nous estimons que 85 % ont été surtraitées et que le diagnostic selon la GSC était incorrect chez 524 (83 %) d'entre elles.

**Conclusions :** Le surtraitement et le diagnostic inexact des infections sur la base du jugement subjectif dans l'approche syndromique des femmes symptomatiques constituent un important gaspillage économique et peuvent en outre contribuer au développement de résistances. Par ailleurs, ces femmes mal diagnostiquées peuvent aussi constituer un réservoir et transmettre des infections à leurs partenaires sexuels.

Translated from English version into French by Suzanne Assenat, through

TRANSLATORS
WITHOUT BORDERS

## Сравнительный анализ синдромальной и ПЦР-диагностики обнаруживает неверное диагностирование/избыточное лечение трихомоноза на основании субъективных оценок пациентов с симптомами данной болезни

Субаш Чандра Сонкар, Кирти Васник, Анита Кумар, Пратима Миттал, Даман Салуджа

**Аннотация**

**Исходные данные.** Влагалищная трихомонада (*Trichomonas vaginalis*) — это одноклеточный организм рода трихомонад, который является возбудителем трихомоноза — заболевания, передающегося половым путем (ЗППП) у мужчин и женщин. Детальное исследование трихомоноза представляет трудности ввиду ограниченных ресурсов, а также применения низкостоимостных диагностических методов с низкой чувствительностью и/или специфичностью. Национальная организация по контролю за СПИДом в Индии (NACO) для лечения рекомендовала использовать синдромальное ведение пациентов (СВП). Цель

настоящего исследования — сравнить полезность алгоритмов NACO-NACP III для лечения ИППП/ИРТ, используемых врачами после постановки диагнозов, основанных на ПЦР.

**Методы.** Участниками исследования, имевшего целью сравнить эффективность ПЦР-диагностики и СВП, стали пациенты акушерско-гинекологического отделения медицинского колледжа «Вардхман Махавир» и больницы «Сафдарджанг» в г. Нью-Дели с января 2011 года до июня 2014 года. На основании СВП, пациентов (820 чел.) лечили антибиотиками при помощи заготовленных наборов для лечения ИППП/ИРТ (инфекций, передающихся половым путем/инфекций репродуктивного тракта; наборы закуплены Национальным обществом по контролю за СПИДом/Государственным обществом по контролю за СПИДом (NACO/SACS) Министерства здравоохранения и планирования семьи Правительства Индии) в рамках Национальной программы по контролю за СПИДом (NACP III) для синдромального ведения пациентов (СВП). От данных пациентов также получены эктоцервикальные сухие мазковые пробы, из которых 634 пробы были проверены в рамках ПЦР-диагностики. Из данных проб была извлечена общая геномная ДНК, которая была использована как образец для ПЦР-амплификации с использованием ген-специфичных праймеров *pfoB, gyrA* и *orf1* для диагностирования организмов *T. vaginalis* (*TV*), *Chlamydia trachomatis* (*CT*) и *Neisseria gonorrhoeae* (*NG*) соответственно.

**Результаты.** Из 6 000 пациентов, которые прошли лечение в акушерско-гинекологическом отделении, 820 (14 %) женщин жаловались на влагалищные выделения; им было рекомендовано лечение антибиотиками против одного или нескольких патогенов, а именно *TV, CT, NG* и *Candid*, или против смешанной инфекции. На основании симптомов и инструкций NACO наблюдалось следующее распределение различных инфекций: *TV* (46 %), *CT* (20 %), смешанная инфекция *TV* и *CT* (12 %), смешанная инфекция *CT* и *NG* (11 %), смешанная инфекция *TV, CT* и *Candida* (7 %) и смешанная инфекция *TV* и *NG* (2 %). Остальные пациенты были инфицированы только *NG* (1 %), полиинфицированы *TV* и *Candida* (0,4 %), а 0,3 % были полиинфицированы *CT, NG* и *Candida*. В результате применения ПЦР-метода 110 (17 %) женщин показали положительный результат на один или более из трех указанных патогенов, а 524 (83 %) женщины показали отрицательный результат на всех три патогена, что, однако, не исключает наличия у них других ИППП, пробы на которые в рамках настоящего исследования не выполнялись. Поскольку всем пациентам (634 чел.) давали антибиотики, мы полагаем, что в отношении 85 % пациентов имело место избыточное лечение, а 524-м пациентам (83 %) в рамках СВП был поставлен неправильный диагноз.

**Выводы.** Избыточное лечение и неточное диагностирование патогенов из-за субъективных оценок пациентов в рамках синдромального ведения женщин с симптомами болезни приводит к значительным материальным растратам и является одной из причин повышения устойчивости к заболеванию. Пациенты, которым поставлен неправильный диагноз, переносят патогены своим половым партнерам.

Translated from English version into Russian by Andriy Lapin, through

TRANSLATORS WITHOUT BORDERS

**El análisis comparativo de ensayos diagnósticos sindrómicos y basados en pruebas de la PCR revela errores de diagnóstico/ sobretratamiento de la tricomoniasis cuando el diagnóstico se basa en una evaluación subjetiva de los pacientes sintomáticos.**

Subash Chandra Sonkar, Kirti Wasnik, Anita Kumar, Pratima Mittal, Daman Saluja

**Resumen**

**Antecedentes:** La *Trichomonas vaginalis* es una enfermedad de transmisión sexual (ETS) que produce la tricomoniasis tanto en hombres como en mujeres. Los métodos diagnósticos de la tricomoniasis constituyen un problema en entornos de bajos recursos ya que los métodos diagnósticos económicos tienen baja sensibilidad y/o especificidad. En India, la Organización Nacional para el Control del SIDA (NACO) recomienda el manejo sindrómico de casos para el tratamiento. El objetivo del presente estudio fue comparar la utilidad de los algoritmos NACO-NACP III para el tratamiento de infecciones de transmisión sexual/ enfermedades del aparato reproductor utilizados por médicos clínicos que se basan en un diagnóstico por PCR.

**Métodos:** Los pacientes que visitaron el Servicio de Obstetricia y Ginecología, en el Vardhman Mahavir Medical College y en el Hospital Safdarjung, en Nueva Delhi entre enero del 2011 y junio del 2014 fueron inscritos en un estudio para comparar la eficacia del diagnóstico en los ensayos en base a PCR y aquellos en base a manejo sindrómico de casos. En el manejo sindrómico de casos, se trató a los pacientes (n=820) con antibióticos utilizando sets pre-empacados para infecciones de transmisión sexual/infecciones del aparato reproductor; provistos por el Control Nacional de SIDA y la Sociedad Estatal de Control del SIDA (NACO/SACS), el Ministerio de Salud y Bienestar Familiar, Gobierno de India. ) bajo el Programa de Control Nacional del SIDA (NACP III) para el manejo sindrómico de casos. También se obtuvieron de estos pacientes muestras de barridos ectocervicales secos y de las mismas 634 fueron analizadas con PCR. De estas muestras se extrajo el ADN genómico total y se lo utilizó como modelo para la amplificación PCR mediante el uso de *pfoB*, *gyrA* y *orf1*, cebadores de gen específicos para el diagnóstico de *T. vaginalis* (TV), *Chlamydia trachomatics* (CT) y *Neisseria gonorrhoeae* (NG) respectivamente.

**Resultados:** De los 6000 pacientes que visitaron OPD, 820 (14%) pacientes mujeres reportaron secreción vaginal y se les recomendó tratamiento antibiótico para uno o más de los patógenos, ya sea *TV, CT, NG* y *Cándida* o para una co-infección. De acuerdo a los signos y síntomas y pautas de NACO, se observó la siguiente distribución de varias infecciones: TV (46%), CT (20%), co-infección de TV y CT (12%), co-infección de CT y NG (11%), co-infección de TV, CT y Cándida (7%) y co-infección de TV y NG (2%). Otros pacientes solo estaban infectados por NG (1%), co-infectados por TV y Cándida (0,4%) y 0,3% estaban infectados por CT, NG y Cándida. De acuerdo al método PCR, 110 (17%) mujeres fueron positivas para uno o más de estos tres patógenos mientras que 524 (83%) de las pacientes fueron erróneamente diagnosticadas mediante el uso de manejo sindrómico de casos. Debido a que a todas las pacientes (634) se les suministró antibióticos, estimamos que el sobretratamiento fue del 85% mientras que 524 (83%) de las pacientes también fueron erróneamente diagnosticadas mediante manejo sindrómico de casos.

**Conclusiones:** El sobre-tratamiento y el diagnóstico erróneo de patógenos como consecuencia de la opinión subjetiva basada en el método sindrómico en mujeres sintomáticas es un desperdicio económico significativo y puede asimismo contribuir hacia un incremento en la resistencia. Las pacientes erróneamente diagnosticadas también sirven como reservorios para la transmisión de patógenos a sus parejas sexuales.

Translated from English version into Spanish by Maria Alejandra Aguada, through

TRANSLATORS WITHOUT BORDERS
